# Thoracic aortopathy in Marfan syndrome overlaps with mechanisms seen in bicuspid aortic valve disease

**DOI:** 10.3389/fcvm.2023.1018167

**Published:** 2023-02-09

**Authors:** Nimrat Grewal, Onur Dolmaci, Evert Jansen, Robert Klautz, Antoine Driessen, Robert E. Poelmann

**Affiliations:** ^1^Department of Cardiothoracic Surgery, Amsterdam University Medical Center, Amsterdam, Netherlands; ^2^Department of Cardiothoracic Surgery, Leiden University Medical Center, Leiden, Netherlands; ^3^Department of Anatomy and Embryology, Leiden University Medical Center, Leiden, Netherlands; ^4^Institute of Biology, Animal Sciences and Health, Leiden University, Leiden, Netherlands; ^5^Department of Cardiology, Leiden University Medical Center, Leiden, Netherlands

**Keywords:** bicuspid aortic valve, Marfan syndrome, aortopathy, pathology, risk stratification

## Abstract

**Background:**

Thoracic aortopathy is a serious complication which is more often seen in patients with Marfan syndrome (MFS) and patients with a bicuspid aortic valve (BAV) than in individuals with a tricuspid aortic valve (TAV). The identification of common pathological mechanisms leading to aortic complications in non-syndromic and syndromic diseases would significantly improve the field of personalized medicine.

**Objective:**

This study sought to compare thoracic aortopathy between MFS, BAV, and TAV individuals.

**Materials and methods:**

Bicuspid aortic valve (BAV; *n* = 36), TAV (*n* = 23), and MFS (*n* = 8) patients were included. Ascending aortic wall specimen were studied for general histologic features, apoptosis, markers of cardiovascular ageing, expression of synthetic and contractile vascular smooth muscle cells (VSMC), and fibrillin-1 expression.

**Results:**

The MFS group showed many similarities with the dilated BAV. Both patient groups showed a thinner intima (*p* < 0.0005), a lower expression of contractile VSMCs (*p* < 0.05), more elastic fiber thinning (*p* < 0.001), lack of inflammation (*p* < 0.001), and a decreased progerin expression (*p* < 0.05) as compared to the TAV. Other features of cardiovascular ageing differed between the BAV and MFS. Dilated BAV patients demonstrated less medial degeneration (*p* < 0.0001), VSMC nuclei loss (*p* < 0.0001), apoptosis of the vessel wall (*p* < 0.03), and elastic fiber fragmentation and disorganization (*p* < 0.001), as compared to the MFS and dilated TAV.

**Conclusion:**

This study showed important similarities in the pathogenesis of thoracic aortic aneurysms in BAV and MFS. These common mechanisms can be further investigated to personalize treatment strategies in non-syndromic and syndromic conditions.

## 1. Introduction

An aortic aneurysm is a pathological dilation of the vessel, which predisposes individuals for life threatening aortic complications such as an acute aortic dissection or a rupture. Mortality in thoracic aortic dissection has been reported as high as 94% (1) and half of patients with a thoracic aortic dissection die before reaching a specialist center. Emergency surgical treatment for patients with an acute aortic dissection is known to have a mortality between 28 and 58% (2, 3). Thoracic aortopathy in tricuspid aortic valve (TAV) individuals is regarded as degenerative and a result of cardiovascular risk factors. However, thoracic aortopathy is predominantly a result of genetic disorders and can therefore develop in young individuals without any signs of cardiovascular ageing (4, 5). Hereditary forms of thoracic aortic aneurysms and dissections are considered non- or syndromic on basis of the absence or presence of symptoms in other organ systems, respectively. A bicuspid aortic valve (BAV) is the most prevalent non-syndromic congenital cardiac disease with a sharply increased risk for thoracic aortic aneurysms and dissections (6). Marfan syndrome (MFS) on the other hand is a common genetic condition, which is highly associated with thoracic aortopathy along with several other clinical features, due to an abnormal development of the connective tissue component fibrillin-1 (7).

As thoracic aortopathy does not have preceding symptoms, the first manifestation often is chest pain due to a ruptured aorta. To prevent lethal aortic complications, patients at risk should be identified and surgically treated in an early stage. Currently, the aortic dimension is the only criterion considered when selecting patients for preventive aortic surgery. Both the European and American aortic surgery guidelines recommend considering surgical replacement of the aorta in bicuspid patients when the diameter reaches 5 or 4.5 cm in a concomitant procedure (8, 9). However, this geometrical cut-off has been a subject of much debate. Many adverse aortic events occur at a diameter below the surgical threshold and some patients might never develop an aortic complication while having a large aneurysm (10, 11). Hence, there is an unmet need to be informed about the intrinsic aortic pathology which predisposes individuals for future lethal aortic events.

As thoracic aneuryms formation in non-syndromic and syndromic conditions is a sliding scale, we need to combine different approaches to the pathogenesis to be able to identify the patients which are susceptible for aortic complications. The identification of common pathological mechanisms in non-syndromic and syndromic conditions and the establishment of a prognostic classification system in the thoracic aortopathy would significantly improve the individual cardiovascular risk stratification and thus result in a major improvement in the field of personalized medicine. The aim of this study is therefore to provide a histopathological comparison of thoracic aneurysms between degenerative, non-syndromic and syndromic conditions in TAV, BAV, and MFS individuals, respectively, and unravel potential common pathways.

## 2. Materials and methods

### 2.1. Ascending aortic wall tissue samples

Non- and dilated ascending aortic wall specimen were collected from BAV and TAV patients, and dilated aortic samples from MFS patients. The aorta was considered dilated if the diameter was 45 mm or more, which is the current cut off for concomitant aortic surgery (12).

Non-dilated TAV (*n* = 11) specimen were obtained post-mortem, from patients deceased from a non-cardiac cause. Non-dilated BAV specimen were available from BAV patients surgically treated with a stentless root replacement (*n* = 11) and six non-dilated BAV samples were obtained from post-mortem donors from the Heart Valve Bank Rotterdam, which were declared unfit for donation due to their BAV.

Dilated BAV, TAV, and MFS specimen were obtained during elective aortic replacement surgery. MFS aortic wall specimen were provided by the AUMC.

The patients were divided into five groups: (1) TAV without dilation (TA, *n* = 11, all obtained post-mortem); (2) TAV with dilation (TAD, *n* = 12, all obtained surgically); (3) BAV without dilation (BA, *n* = 17; of which 11 obtained surgically and six post-mortem); (4) BAV with dilation (BAD, *n* = 19, all obtained surgically); and (5) MFS with dilation (MFS, *n* = 8, all obtained surgically).

We know from our previous studies that BAV patients with an aortic valve stenosis have an increased cardiovascular risk profile as compared to BAV patients with a regurgitant aortic valve (13, 14). We therefore included all BAV and TAV patients undergoing surgery with a severe aortic valve stenosis with either mild or no regurgitation. The BAV and TAV patients thus had comparable valve pathology and an underlying increased cardiovascular risk profile.

Sample collection was uniform in all patients: ascending aortic wall specimen were obtained from the aortotomy site. The aortotomy site is classically in the middle of the ascending aorta, just beneath the aortic fat pad. Circular tissue was obtained and embedded in paraffin. The complete circular ascending aortic wall was sectioned to avoid sampling of the aortic tissue.

The process of formalin fixation, decalcification, and embedding has been described in our previous publications (15).

### 2.2. Ethical approval

Sample collection and handling was carried out according to the official guidelines of the Medical Ethical Committee of the Leiden University Medical Center (LUMC), Leiden, the Medical Ethical Committee of the Amsterdam University Medical Center (AUMC), Amsterdam, and the code of conduct of the Dutch Federation of Biomedical Scientific Societies.^1^ The Medical Ethical Committee approved the inclusion of these specimen in this study. Written informed consent was obtained for inclusion in the study.

The Heart Valve Bank Rotterdam provided us with aortic tissue from six patients with a BAV. The specimen were obtained from post-mortem donors, which were declared unfit for donation due to their BAV. The Advisory Board of the Heart Valve Bank approved the inclusion of these specimen in this study.

### 2.3. Histology and immunohistochemistry

To study the histomorphology of the vessel wall, the sections were stained with hematoxylin-eosin (HE), resorcin fuchsin (RF), and Movat pentachrome staining. Vascular smooth muscle cell expression was studied with differentiated vascular smooth muscle cell markers: alpha smooth muscle actin (αSMA), smooth muscle 22 alpha (SM22α), and smoothelin. Lamin A/C was studied as a marker for myoblast differentiation. Progerin, which is a splice variant of lamin A/C, was studied as a marker of cardiovascular aging (16).

The staining protocols for immunohistochemistry have been previously described (17).

The primary antibodies were used against αSMA (1/5000, A2547, Sigma-Aldrich Chemie), SM22α (1/100, AB10135, Abcam), smoothelin (1/200, 16,101, Progen Biotechnik), lamin A/C (1/100, MAB3211, Millipore), progerin (1/50, SC-81611, Bio-Connect), cleaved caspase-3 (1/250, 9,661, Cell Signaling), and fibrillin-1 1/100 (MAB1919, Millipore). The secondary antibodies used were peroxidase-conjugated rabbit anti-mouse 1/250 (DAKO p0260) for αSMA, goat anti-rabbit biotin 1/200 (Vector Laboratories, United States, BA-1000) and goat serum 1/66 (Vector Laboratories, United States, S1000) for cleaved caspase-3, SM22α, and progerin, and horse anti-mouse biotin 1/200 (Santa Cruz Biotechnology, Inc., CA, United States, SC-9996-FITC) in horse serum 1/66 (Brunschwig Chemie, Switzerland, S-2000) for smoothelin, lamin A/C, and fibrillin-1.

### 2.4. Histological, immunohistochemical, and morphometric analysis

Sections were studied with a Leica BM5000 microscope equipped with plan achromatic objectives (Leica Microsystems, Wetzlar, Germany).

Morphology of the vessel wall was studied with hematoxylin-eosin, resorcin fuchsin (RF), and Movat pentachrome staining. The aortic wall was studied in a standardized way using the grading system described in the aortic consensus paper statement on surgical pathology of the aorta (18, 19). Overall medial degeneration (OMD), elastic fiber fragmentation and loss (EFF/L), elastic fiber thinning (EFT), elastic fiber disorganization (EFD), mucoid extra cellular matrix accumulation (MEMA), and smooth muscle cell nuclei loss (SMCNL), were studied in all sections and indexed from zero (none), one (mild), two (moderate), to three (severe) on three predetermined locations (left, middle, and right) of every section, that we refer to as “microscopic fields” maintained in evaluation of all stainings on sister sections. Additionally, aortic adventitial inflammation was quantified, indexed from zero (no inflammatory cells), one (a few cells), two (groups of cells) to three (large clusters of cells). The maximum intimal thickness was quantified as the maximal distance in micrometers between the endothelial layer lining the luminal surface and the first major internal elastic lamella, excluding atherosclerotic areas.

The cytoplasmatic level of expression of αSMA, SM22α, and smoothelin, intra- and extracellular expression of fibrillin-1 and nuclear expression of lamin A/C, progerin and cleaved-caspase-3, were analyzed similarly. In each microscopic field, the level of expression was indexed for αSMA, SM22α, smoothelin, and fibrillin-1 as zero (no expression in the media layer), one (expression in less than one third of the medial layer), two (expression in two thirds of the medial layer), and three (expression in the whole medial layer). To determine the level of lamin A/C, progerin, and caspase-3 expression, the number of positively stained nuclei was counted and analyzed using ImageJ in the three fields for each stained section. A threshold was applied to filter background noise. The total number of cells (positively and negatively stained nuclei and cytoplasm) was not different between specimens. Therefore, in each microscopic field, the number of lamin A/C, progerin, and caspase-3 positive cells was normalized to the total number of cells per 10^5^ μm^2^. To quantify the number of positive cells, the incidence of positive cells and signal intensity were measured using ImageJ. Finally, the number of normalized positive cells for each staining was averaged between the three microscopic fields.

Re-evaluation of all samples was performed by an independent, experienced histopathologist who was blinded to the clinical data, and who confirmed the findings.

### 2.5. Statistical analysis

All numerical data are presented as the mean and standard deviation of three microscopic fields on each stained slide. Statistical differences between all five groups (*non-dilated and dilated TAV, non-dilated and dilated BAV and MFS*) were analyzed with the one, two, and three-way ANCOVA tests. *Post-hoc* analysis was performed in variables with a significant difference. The Mann–Whitney *U* test was used for comparing continuous variables (with a not normally distribution) within the groups separately. Comparisons were corrected for age with a regression analyses. Significance was assumed when *p* < 0.05 with SPSS 25.0 (SPSS Inc. Chicago, Ill) was used for the statistical analyses. *Graphs are prepared with Graphpad Prism*.

## 3. Results

### 3.1. Patient characteristics

Patients included in this study were divided in five groups shown in Table 1. Marfan patients were statistically the youngest, followed by the non- and dilated BAV patients (*p* < 0.001; Table 1). The BAV groups showed a predominant male gender, whereas in the MFS female and male were equally present.

**Table 1:** Patient characteristics.

Characteristic	TA	TAD	BA	BAD	MFS	Value of *p*
Number of patients	11	12	17	19	8	
Specimen obtained	Postmortem	Surgically	Postmortem *n* = 6 Surgically *n* = 11	Surgically	Surgically	
Age (years)	64.5 ± 9.0	72.3 ± 11.2	55.8 ± 9.8	60.8 ± 7.6	36.1 ± 11.8	<0.001
Male (%)	6 (54.5)	4 (33.3)	12 (70.6)	16.(84.2)	3 (37.5)	0.025

TA, non-dilated tricuspid aortic valve; TAD, dilated tricuspid aortic valve; BA, non-dilated bicuspid aortic valve; BAD, dilated bicuspid aortic valve; and MFS, dilated Marfan syndrome.

### 3.2. Cardiovascular ageing and pathology score features

**Figure 1:**
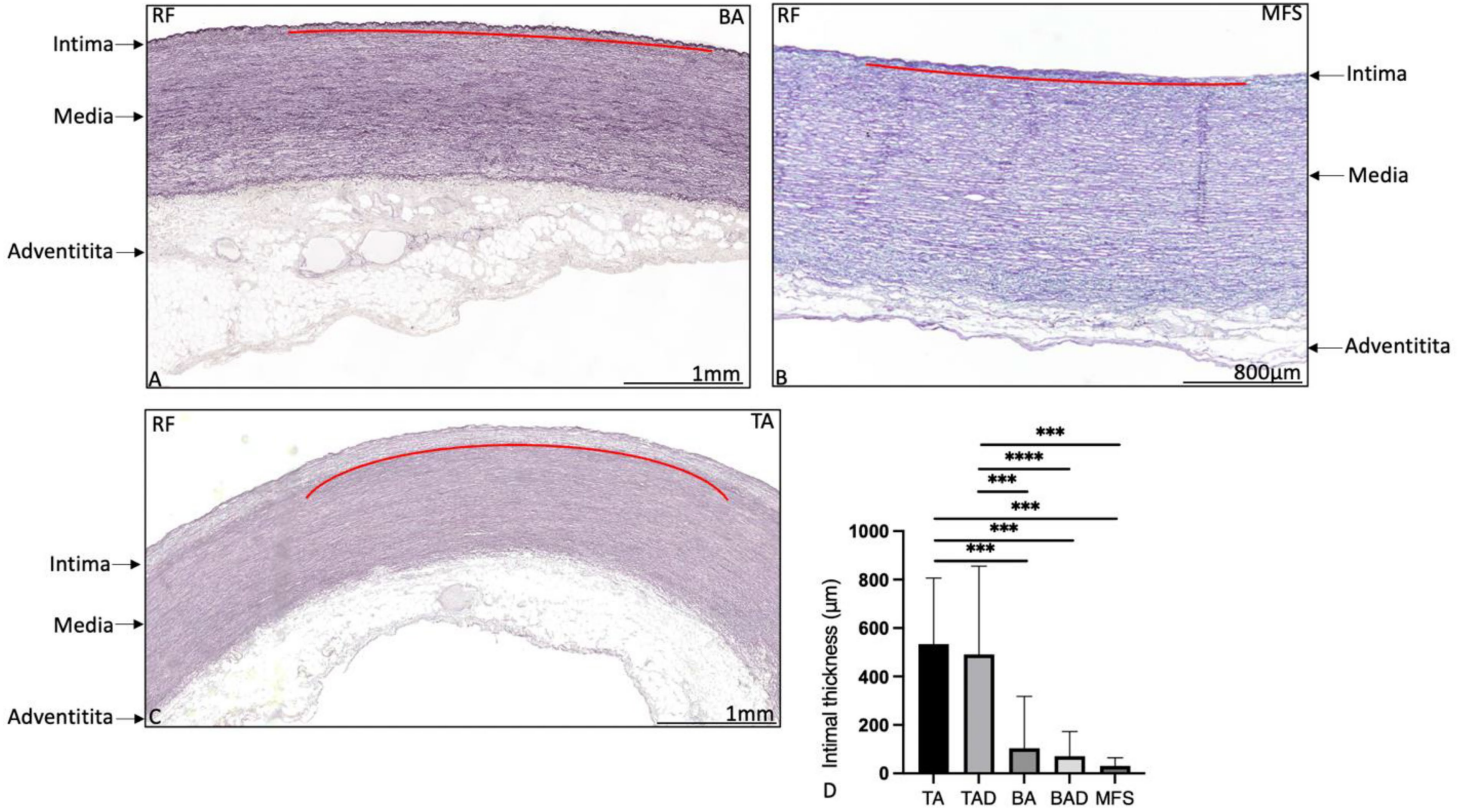
Transverse histologic sections of the ascending aortic wall in a non-dilated bicuspid aortic valve patient **(A)**, in a Marfan syndrome patient **(B)** and in a non-dilated tricuspid aortic valve patient (**C**; 5 μm) stained with resorcin fuchsin. The intimal, medial, and adventitial layer are depicted with an arrow in the figure. The intimal layer is indicated with a red line in **(A–C)**. The intimal layer is formed by a single layer of a lamina elastica interna in the bicuspid **(A)** and Marfan patient **(B)**, whereas the intima is significantly thicker in the tricuspid aortic valve patient **(C)**, graph **(D)**. Elastic lamellae are fine and neatly organized without fragmentation or degeneration. BA, non-dilated bicuspid aortic valve; MFS, Marfan syndrome; TA, non-dilated tricuspid aortic valve; and RF, resorcin fuchsin, ***p<0.001. Scale bar is shown in the figures.

**Figure 2:**
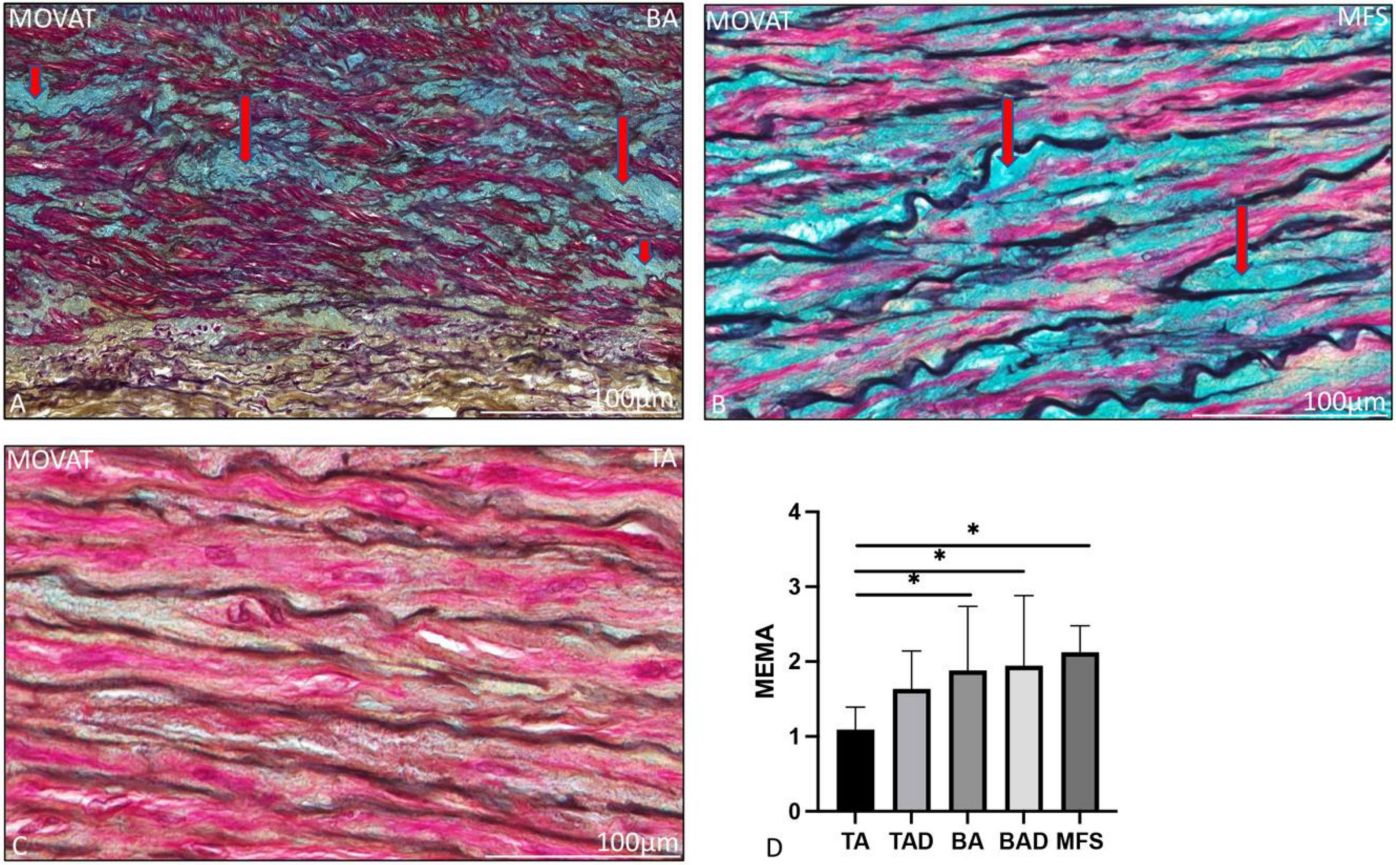
Transverse histologic sections of a non-dilated dilated bicuspid **(A)**, Marfan syndrome **(B)** and a non-dilated tricuspid ascending aortic wall (**C**; 5 μm) stained with MOVAT pentachrome staining. **(A)** shows mucoid extracellular matrix accumulation which stains light blue (indicated with the red arrows) in the MOVAT pentachrome staining, the vascular smooth muscle cells are red, elastic fibers are seen in dark purple, collagen and reticulin in yellow, and nuclei in black. Mucoid extracellular matrix accumulation was significantly greater in the bicuspid **(A)** and Marfan patients **(B)** as compared to the non-dilated tricuspid aortic valve group **(C)**, graph **(D)**. BA, non-dilated bicuspid aortic valve; BAD, dilated bicuspid aortic valve; MEMA, mucoid extracellular matrix accumulation; MFS, Marfan syndrome; TA, non-dilated tricuspid aortic valve; and TAD, dilated tricuspid aortic valve, *p<0.05. Scale bar is shown in the figures.

**Figure 3:**
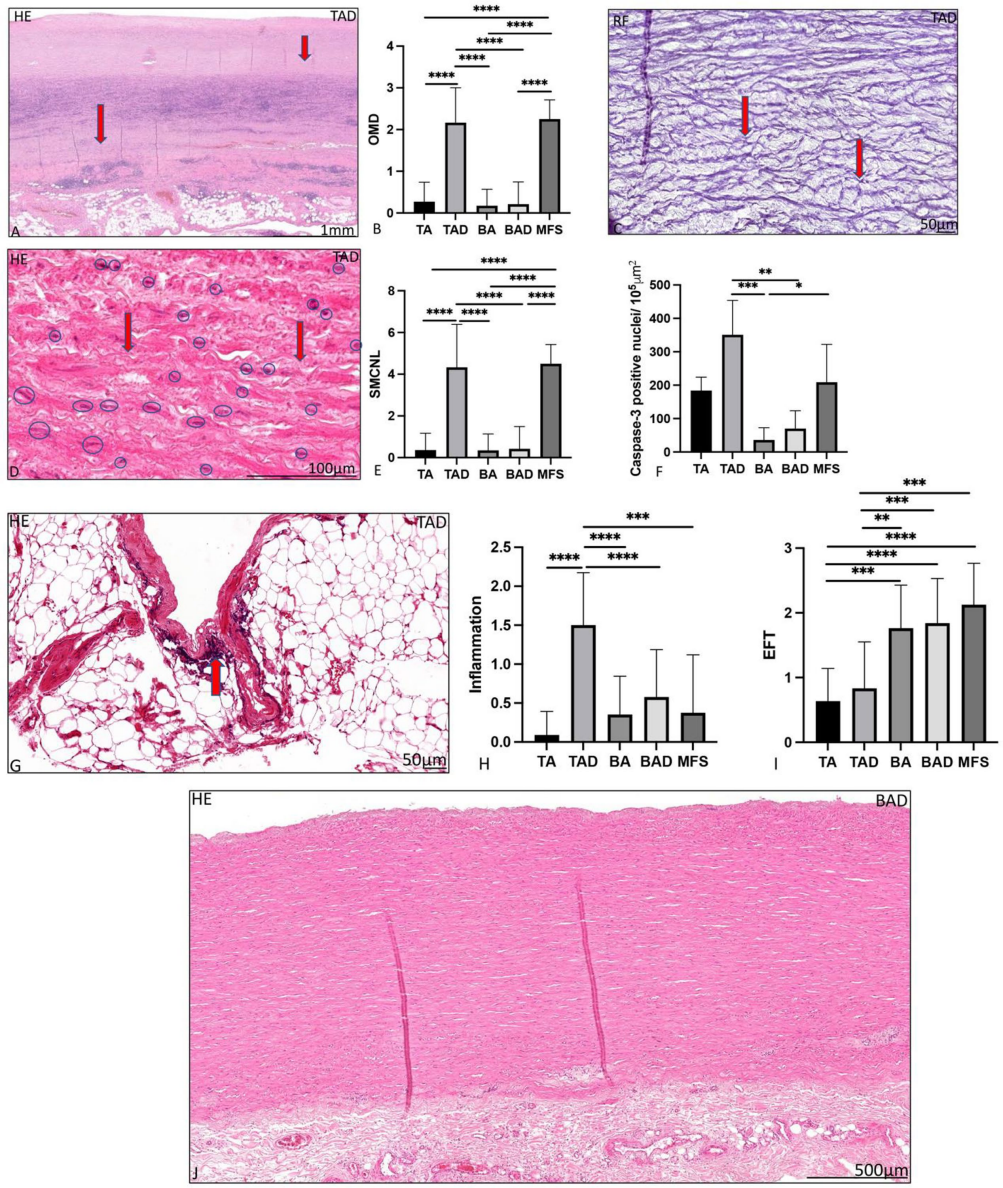
Transverse histologic sections of a dilated tricuspid ascending aortic wall **(A,C,D,G)** and a dilated bicuspid aortic valve (**J**; 5 μm) stained with hematoxylin eosin **(A,D,G,J)**, resorcin fuchsin **(C)**. **(A)** shows overall medial degeneration, which is indicated with the red arrows. Overall medial degeneration was significantly greatest in the dilated tricuspid aortic valve and Marfan syndrome group, graph **(B)**. Elastic fiber fragmentation is indicated with the red arrows in **(C)**. In **(D)**, the vascular smooth muscle cells are surrounded by a blue circle, the areas with loss of smooth muscle cell nuclei are indicated with an arrow. Smooth muscle cell nuclei loss was significantly greatest in the dilated tricuspid aortic valve and Marfan syndrome groups, graph **(E)**, which was confirmed with the caspase-3 staining shown in **(F)**. Elastic fiber thinning was significantly greatest in the bicuspid and Marfan groups **(I)**. In **(J)** in comparison a dilated ascending aortic wall is shown in the bicuspid aortic valve without features of overall medial degeneration, smooth muscle cell nuclei loss and adventitial inflammatory cells. BA, non-dilated bicuspid aortic valve; BAV, bicuspid aortic valve; EFF/L, elastic fiber fragmentation and loss; EFT, elastic fiber thinning; HE, hematoxylin eosin; MEMA, mucoid extracellular matrix accumulation; OMD, overall medial degeneration; RF, resorcin fuchsin; SMCNL, smooth muscle cell nuclei loss; TA, non-dilated tricuspid aortic valve; and TAV, tricuspid aortic valve, *p<0.05, **p<0.01, ***p<0.001, **** *p*<0.0001. Scale bar is shown in the figures.

The ascending aortic wall is divided in three layers, the tunica intima, media, and adventitia (Figure 1). Pathology score features are shown in Figures 2, 3.

The non-dilated BAV and TAV ascending aortic wall exhibited minimal medial pathology. Features which were found significantly distinct between these two groups were mucoid extracellular matrix accumulation and elastic fiber thinning which were both more profound in the non-dilated BAV as compared to the non-dilated TAV (*p* = 0.01 and *p* < 0.05, respectively, Figure 2). All non-dilated MFS and BAV patients had a significantly thinner intimal layer as compared to all TAV patients (*p* < 0.001; Figure 1).

The dilated MFS group had many pathological features of the aortic media in common with the dilated TAV group, which were significantly profound as compared to the non-dilated BAV group including overall medial degeneration (*p* < 0.0001; Figures 3A,B), elastic fiber fragmentation and loss (*p* < 0.001; Figure 3C), elastic fiber disorganization (*p* < 0.001), and smooth muscle cell nuclei loss (*p* < 0.0001; Figures 3D–F). Smooth muscle cell nuclei loss in the MFS and dilated TAV was confirmed by studying the expression of caspase-3 (*p* = 0.03; Figure 3F). Regression analysis showed a significant difference in the cardiovascular ageing marker progerin levels between the TAD and BAD groups (*p* = 0.048), which remained significant after correcting for the difference in age [OR 1.02 (95% CI 1.001–1.038); *p* = 0.036].

The dilated MFS also had some histopathological features in common with the dilated BAV, including lack of inflammatory cells in the adventitia and thinning of the elastic fibers, both significantly distinct from the TAV group (*p* < 0.001 and *p* = 0.001, respectively, Figures 3G–I). The dilated MFS and BAV patients also had a significantly thinner intimal layer as compared to the dilated TAV patients (*p* < 0.001; Figure 1). Mucoid extracellular matrix accumulation was not found different between the three dilated aortic specimen groups (Figure 2D).

### 3.3. Vascular smooth muscle cell expression and fibrillin-1

**Figure 4:**
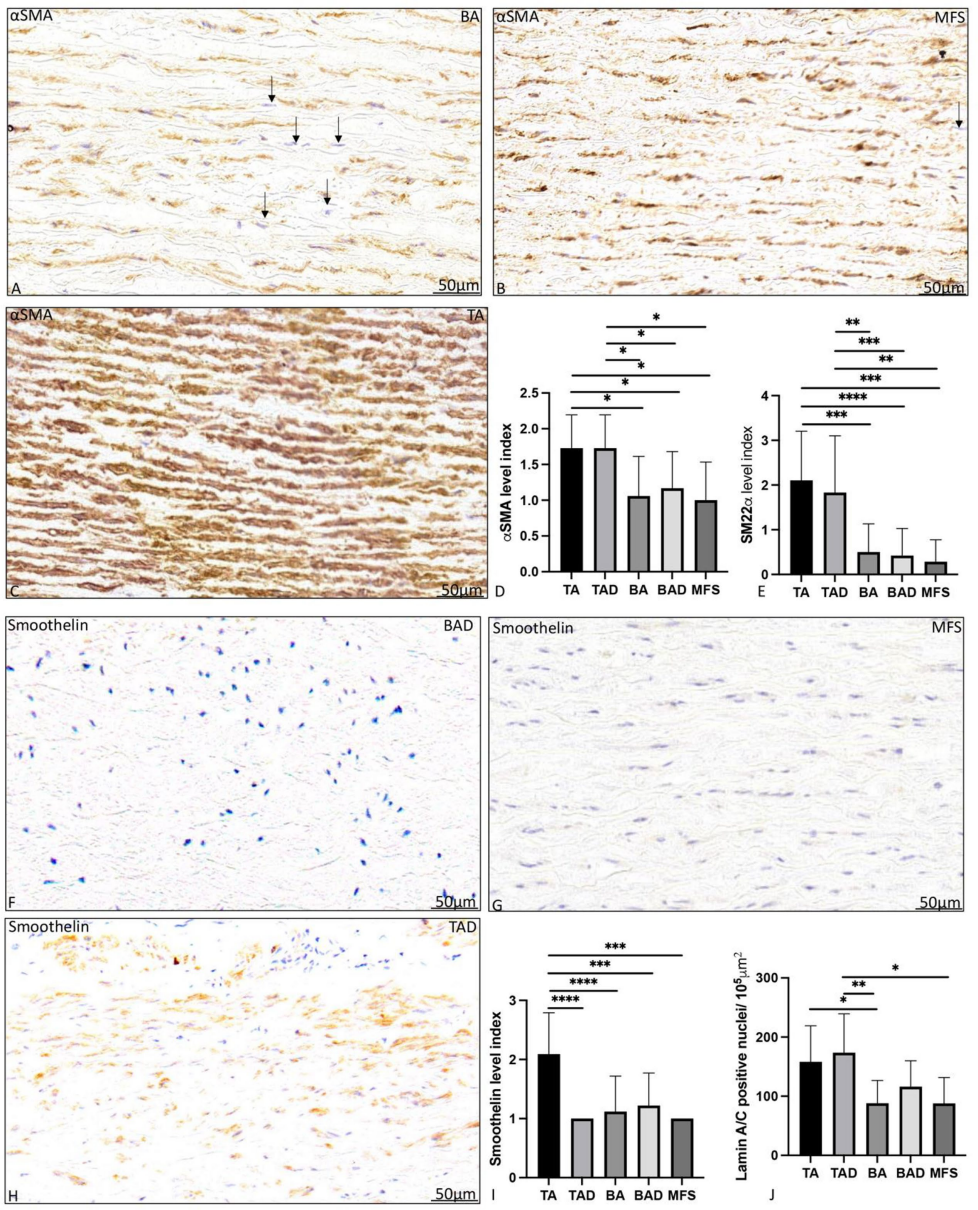
Transverse histologic sections of a non-dilated ascending aortic wall in bicuspid aortic valve **(A)**, Marfan syndrome **(B)** and tricuspid aortic valve **(C)**; and a dilated ascending aortic wall in bicuspid aortic valve **(F)**, Marfan syndrome **(G)** and tricuspid aortic valve (**H**; 5 μm); stained with alpha smooth muscle actin **(A–C)** and smoothelin **(F–H)**. Alpha smooth muscle actin expression was significantly highest in the non- and dilated tricuspid aortic valve patients **(A–D)**. Level of expression was lower in the bicuspid and Marfan patients as individual cells expressed less alpha smooth muscle actin as pointed out with the arrows highlighting cells (blue nuclei) which are devoid of expression. Smooth muscle 22 alpha expression was also significantly higher in the tricuspid aortic valve groups as compared to the bicuspid and Marfan patients **(E)**. Smoothelin expression was significantly highest in the non-dilated tricuspid aortic valve patients **(F–I)**. Lamin A/C expression is shown in (Figure 5E) with significantly highest expression the non-dilated and dilated tricuspid patients. αSMA, alpha smooth muscle actin; BA, non-dilated bicuspid aortic valve; BAD, dilated bicuspid aortic valve; SM22α, smooth muscle 22 alpha; TA, non-dilated tricuspid aortic valve; TAD, dilated tricuspid aortic valve, *p<0.05, **p<0.01, ***p<0.001, **** *p*<0.0001. Scale bar shown in the figures.

Non-dilated BAV patients had a significantly lower expression of vascular smooth muscle cell markers αSMA, SM22α, and smoothelin as compared to the non-dilated TAV patients (*p* = 0.05, *p* < 0.001, and *p* = 0.002, respectively, Figure 4).

aDilated MFS and BAV patients showed a significantly lower expression of vascular smooth muscle cells markers αSMA (*p* = 0.02, *p* = 0.013, respectively, graph 4D) and SM22α (*p* < 0.05, graph 4E) as compared to the dilated TAV patients. Smoothelin expression was almost absent in the dilated MFS, BAV, and TAV group (Figures 4F–I). The level of expression of myofibroblast differentiation marker lamin A/C was also significantly lower in the MFS and BAV as compared to the TAV (*p* < 0.05).

**Figure 5:**
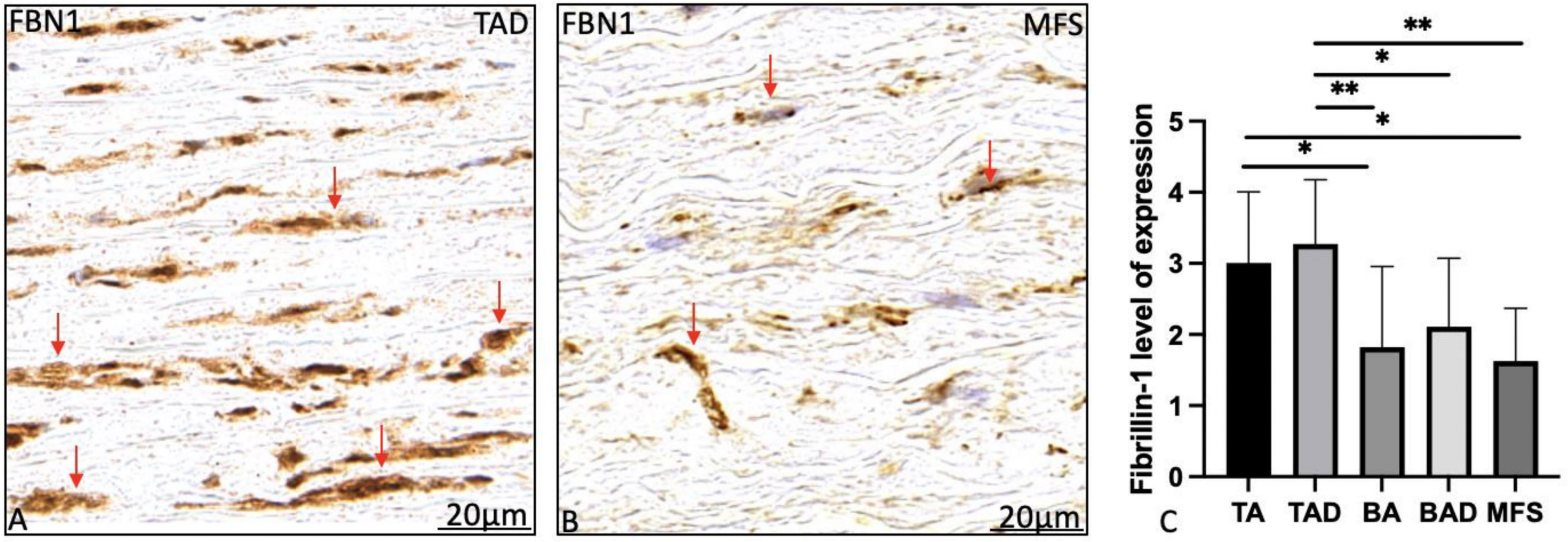
Transverse histologic sections of a dilated tricuspid **(A)** and Marfan (Figure 4B) ascending aortic wall (5 μm); stained with fibrillin-1. Fibrillin-1 expression was significantly highest in the tricuspid aortic valve patients as compared to the Marfan and bicuspid patient groups **(A–C)**. Expression was found extracellular in the tricuspid aortic valve patients (red arrows in **A**) as compared to the intracellular expression in the Marfan patients (red arrows in **B**). BA, non-dilated bicuspid aortic valve; BAD, dilated bicuspid aortic valve; FBN1, fibrillin-1; TA, non-dilated tricuspid aortic valve. TAD, dilated tricuspid aortic valve, *p<0.05, **p<0.01. Scale bar shown in the figures.

Fibrillin-1 expression was significantly lower in the non-dilated BAV as compared to the non-dilated TAV patients (*p* = 0.04; Figure 5). Expression of fibrillin-1 was significantly lower in the dilated MFS and BAV groups as compared to the dilated TAV (*p* = 0.001, *p* = 0.015, respectively, graph 5C). Distribution and localization of fibrillin-1 was intracellular in the BAV and MFS, whereas it was mostly extracellular in the TAV patients as shown in Figure 5.

**Figure 6:**
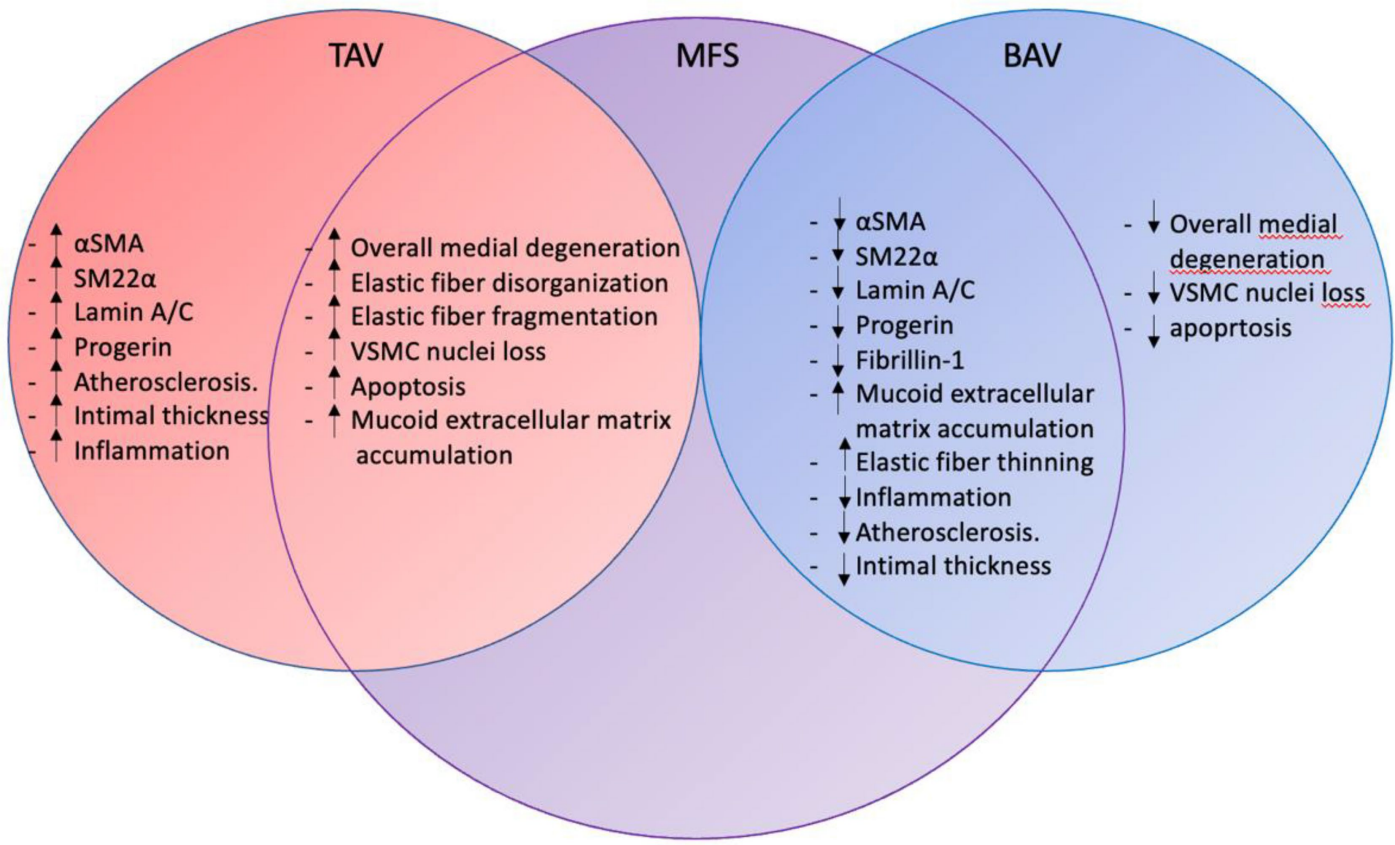
Ascending aortic wall pathology in TAV, MFS, and BAV. The figure summarizes the intimal, medial and adventitial pathology in tricuspid aortic valve, Marfan syndrome and bicuspid aortic valve patients. Marfan syndrome pathology overlaps with mechanisms seen in the tricuspid and bicuspid aortic valve. TAV, tricuspid aortic valve; MFS, Marfan syndrome; BAV, bicuspid aortic valve; αSMA, alpha smooth muscle actin; SM22α, smooth muscle 22 alpha; and VSMC, vascular smooth muscle cell.

Figure 6 summarizes the ascending aortic wall pathology observed in the Marfan, bicuspid, and tricuspid aortic valve patients.

## 4. Discussion

An aortic dissection is the most devastating complication of thoracic aortic disease. Though thoracic aortopathy is a potentially life-threatening event, it is preventable if individuals-at-risk are identified timely and properly managed. Current risk stratification is solely based on a geometric cut off. Recent studies have however emphasized the inaccuracy of this parameter to early detect aortic complications (10, 11). Therefore, there is urgent need to develop a patient tailored risk stratification model to identify patients prone for aortopathy. We postulate that thoracic aortopathy is a homogeneous clinical presentation of heterogeneous underlying deficits sharing common genetic associations, clinical characteristics, and diagnostic and therapeutic challenges. The identification of common pathological mechanisms and effector pathways in the different underlying non-syndromic and syndromic diseases, which lead to thoracic aortopathy would significantly improve this individual aortopathy risk stratification and thus result in a major improvement in the field of personalized medicine.

Bicuspid aortic valve and Marfan syndrome belong to the most common non-syndromic and syndromic conditions which carry an increased risk to develop a thoracic aortic aneurysm and/or dissection. This study aimed at unraveling common pathogenetic mechanisms in syndromic, non-syndromic, and degenerative thoracic aortic aneurysms by studying the ascending aortic wall in MFS, BAV, and TAV, respectively. We defined degenerative medial pathology as the presence of pathology score features and a significant expression of progerin. In our earlier studies, we have paid particular attention to the role of shear stress on the histopathology of the aortic wall in patients with a bicuspid and tricuspid aortic valve, with and without dilatation and studied the pathological features in the ascending aortic wall (20). With magnetic resonance imaging, the area with maximum shear stress in the ascending aortic wall was identified and obtained during surgery. No differences were found in the pathological features between the jet and non-jet side (17). In this study, the role of hemodynamics was therefore not taken into account.

Periaortic inflammation, intimal atherosclerosis, overall medial degeneration, elastic fiber fragmentation, elastic fiber disorganization, vascular smooth muscle cell loss, apoptosis, and mucoid extracellular matrix accumulation were characteristic for the degenerative tricuspid aortic valve patients as has also earlier been described in the literature (21).

Syndromic Marfan patients had some medial pathological features in common with the degenerative tricuspid aortopathy. Most overlap in pathogenesis was however observed between the Marfan and bicuspid patients. Both conditions have an increased risk to develop thoracic aortopathy at a younger age as compared to the tricuspid aortic valve patients. The ascending aortic wall in the non-dilated MFS and BAV showed less differentiated vascular smooth muscle cells and increased amounts of mucoid extracellular matrix between the fine elastic lamellae.

The intimal layer was significantly thinner in both the non-syndromic and syndromic conditions, with scarce amount of atherosclerosis. Periaortic inflammation was also less apparent.

It has earlier been concluded that even though both the intimal and adventitial layers contribute to the disease process (22), progressive mechanical weakening of the aorta is mainly caused by degeneration of the medial layer, regardless of the etiology (14, 23–25). In the current study, medial pathology was also worse as compared to the intimal and adventitial layer in all aortopathy patients. Nevertheless, the pathogenetic features which could lead to mechanical weakening of the aortic media were distinct between the degenerative and genetic causes. In the tricuspid aortic valve patients, features of cardiovascular ageing were predominantly seen in the aortic media. Despite being the youngest patients among the entire study population, the aortic media in MFS was also characterized by similar cardiovascular ageing features including overall medial degeneration, vascular smooth muscle cell apoptosis, and degradation of the elastic lamellae. We compared differences in progerin expression between all groups and found that MFS patients had a significantly lower expression of progerin as compared to the dilated TAVs, the results remained significant after correcting for age indicating that the observed pathology in this group is not the result of accelerated cardiovascular ageing. The BAV on the other hand did not show any signs of degenerative medial pathology. In the MFS and BAV the main medial pathologic feature was a significantly lower expression of differentiated vascular smooth muscle cells in the ascending aortic wall. Medial vascular smooth muscle cells play a key role in the pathogenesis of aneurysm formation. In the adult healthy aorta, the smooth muscle cells are contractile and regulate the vascular tone as their primary function. The smooth muscle cells do not lose their plasticity and can undergo a phenotypic switch from a contractile to a synthetic phenotype. The synthetic vascular smooth muscle cells have an elevated synthetic and proliferative activity resulting in a downregulation of contractile proteins, and an increased deposition of dysfunctional matrix and production of matrix degrading enzymes (20, 26–29). The degradation of the extracellular matrix can result in the activation of several signaling pathways which in turn leads to medial degeneration and progressive weakening of the vessel wall (30). The phenotypic switch is triggered by inflammation and vascular injury (29, 31–33). The local environmental factors that modulate vascular smooth muscle cell phenotype include growth factors, such as platelet-derived growth factor (34, 35) and TGF-β (34, 36), angiotensin II (37), nitric oxide (34), reactive oxygen species (38), and other components of the ECM.

The MFS and BAV also had a significantly lower expression of fibrillin-1. Fibrillin-1 is produced by differentiated quiescent vascular smooth muscle cells (39). In the BAV therefore a decrease in fibrillin-1 production is possible without a FBN-1 mutation, as the ascending aortic wall comprises of immature, synthetic vascular smooth muscle cells. In the MFS a decrease in fibrillin-1 expression is caused by two mechanisms: firstly a FBN-1 mutation causes a decrease in structurally normal fibrillin-1, moreover the immature vascular smooth muscle cells could cause a secondary decrease in the (structurally normal) expression of fibrillin-1. Besides a decreased expression of fibrillin-1in the aortic wall in BAV and MFS, we found that the distribution and localization of fibrillin-1 was different in the MFS and BAV as compared to the TAV. In this paper, we found a reduction in extracellular fibrillin in MFS and BAV and more expression within the vascular smooth muscle cells. This is in line with earlier findings that individuals with MFS associated with cardiovascular complications have a significant reduction in fibrillin deposition with or without a defect in synthesis (40).

In bicuspidy, it has earlier been postulated that thoracic aortopathy is caused by an early embryonic defect in the progenitor cells which are responsible for both the aortic valve and vascular smooth muscle cells development (41). In MFS, the link between a FBN1 mutation and early embryonic defects is less well described. However, many features observed in both the MFS and BAV hint at an early developmental defect, most likely in neural crest-derived smooth muscle cells, which could increase the susceptibility for future aortic complications. Not only are the vascular smooth muscle cells less well differentiated in the aortic media, the intimal layer was also significantly thinner in the MFS and BAV. The intima is formed during embryogenesis and generally increases in thickness in the first years after birth (42). The possibility to adapt the intimal thickness to shear stress is an important aspect which the MFS and BAV lack, although the thinner intima might be protective for atherosclerosis during their life. Lack of atherosclerosis in bicuspid aortopathy has earlier been described clinically (43, 44). It is interesting to note that a specific group of animals, being reptiles, normally present with bicuspid semilunar valves. Evidently, these have a different physiology, being “cold-blooded” as well as anatomy, as they present two aortas. Nevertheless, here the bicuspid valves function efficiently, even until advanced ages (45).

In conclusion, this study summarizes a novel approach to investigate similarities between non-syndromic and syndromic thoracic aortopathy. Currently, patients at risk for future thoracic aortopathy cannot be distinguished and the aortic diameter serves as the sole criterion for aortic surgery. There is an unmet need to for a personalized approach to identify patients at risk for aortic complications on basis of pathological features. The identified common effector pathways in bicuspidy and Marfan syndrome patients could serve as leads to further develop specific disease models for personalized identification and treatment high risk patients.

### 3.1. Study limitations

We designed our study by comparing the expression of a panel of markers distinguishing cases of non- and dilated ascending aortic wall specimen from BAV and TAV patients, and dilated aortic samples from MFS patients. We could not correlate our findings of immunohistochemistry with other techniques such as western blotting as we did not have frozen tissue samples of all the aortic wall specimens, we received fixed in formalin from the various groups.

## Data availability statement

The raw data supporting the conclusions of this article will be made available by the authors, without undue reservation.

## Ethics statement

The studies involving human participants were reviewed and approved by sample collection and handling was carried out according to the official guidelines of the Medical Ethical Committee of the Leiden University Medical Center (LUMC), Leiden, the Medical Ethical Committee of the Amsterdam University Medical Center (AUMC), Amsterdam. Written informed consent was obtained. Six cryopreserved bicuspid human aortic valves were obtained from the Heart Valve Bank in Rotterdam (Erasmus University Medical Center, Rotterdam) originating from post-mortem donors. These valves were declared unfit for implantation because of the bicuspid nature of the valves. The Advisory Board of the Heart Valve Bank allowed these valves to be included in the present project because the research was in line with the permission of the donation. The patients/participants provided their written informed consent to participate in this study.

## Author contributions

NG conceived and designed the experiments, performed the experiments, analyzed and interpreted the data, contributed reagents and materials, and wrote the paper. OD, EJ, and AD wrote the paper. RK conceived and designed the experiments, contributed reagents and materials, and wrote the paper. RP conceived and designed the experiments, analyzed and interpreted the data, contributed reagents and materials, and wrote the paper. All authors contributed to the article and approved the submitted version.

## Conflict of interest

The authors declare that the research was conducted in the absence of any commercial or financial relationships that could be construed as a potential conflict of interest.

## Publisher’s note

All claims expressed in this article are solely those of the authors and do not necessarily represent those of their affiliated organizations, or those of the publisher, the editors and the reviewers. Any product that may be evaluated in this article, or claim that may be made by its manufacturer, is not guaranteed or endorsed by the publisher.

## Footnotes

^1^ www.FMWV.nl
